# The Care of the Insane

**Published:** 1905-05-06

**Authors:** 


### The Hospital.

A Journal of

The Medical Sciences and Hospital Administration.

Vol. XXXVIII.—No. 971. SATURDAY, MAY 6, 1905.

### The Care of the Insane.

The current number of the *Quarterly Review* contains a highly interesting article on the subject of the Care of the Insane. The writer traces the steps by which the charge of idiots and lunatics, originally vested in the Crown, came to be deputed to the Lord Chancellor; and how, mainly in consequence of this history, it has remained to a very large extent in the hands of lawyers, to the practical exclusion of the profession to which it more naturally belongs. He explains the totally unjustifiable distinction which is still drawn between those of the insane who have property and those who have none; the general result being that two medical visitors in lunacy are required to inspect 718 patients, while three medical commissioners are required to fulfil the same duties in relation to 120,000. Partly on this basis, but chiefly on the ground that insanity is now universally recognised to be disease, the reviewer criticises the whole composition of the existing commission with great force and reasonableness. He points out that this composition has been brought about partly by the fact that there is a small but absolute need for legal aid, because the law safeguards those who must be under medical care, partly because throughout the Middle Ages insanity was not regarded as a disease but as demoniacal possession, a delusion which to some extent held its ground even in 1845, when the Lunacy Act was passed; and perhaps most of all by the circumstance that the official head of lunacy is the Lord Chancellor, a high official not likely to be entirely independent of the prejudices which induced the currier in the fable to recommend leather as the best material for fortification. He quotes Sir John Tuke's speech in the House of Commons, a twelvemonth ago, as giving the best possible account of the working of the existing system, under which the medical and legal Commissioners hunt in couples, the legal Commissioner knowing no more of medicine or of lunacy than Policeman X. The idea was, said Sir John, that some point of law might arise in the course of the visit which could only be settled by the legal Commissioner; but he challenged the Commissioners to adduce a single case within the last forty years in which such an emergency had arisen. The general result of the present method of working was that the legal Commissioner wrote the report, and, in fact, performed the duties of a clerk to his medical colleague. On the basis of such facts and considerations as these, the reviewer rests a demand for a very considerable increase in the present medical strength of the Commission, while declaring that any increase to its legal strength would be a waste of the taxpayer's money, and would not be attended with any sort of benefit to the insane.

It will probably be conceded by all who have any practical acquaintance with the subject that the line taken by the reviewer is so far unassailable, and that the only impediments to an urgently required reform must be looked for in the general indifference of politicians to questions which cannot be so manipulated as to serve the interests of party. On this ground, and on this ground alone, it is quite conceivable that a Bill for the amendment of the Lunacy Acts, if introduced into Parliament, would be talked out or dropped out, and that nobody would care. The incident by which the late Mr. Plimsoll carried an Act for the protection of the lives of sailors against the dead weight of official indifference is not likely to be repeated, and it may be assumed that nothing less forcible would suffice for the protection of the insane. But the reviewer is not content with his condemnation of the present methods of supervision and inspection, and proceeds to consider the claims of those so-called "border-land" cases which he declares to be numerous, and for the care and treatment of which the present law not only makes no provision, but even interposes practically insurmountable difficulties. He declares the great want of the day, in this respect, to be for the adoption of a system such as that which exists in Scotland, by which a person with incipient insanity, who is not dangerous to himself or others, can be received for treament for six months on a simple medical certificate that there is a prospect of recovery.

We are tempted to ask whether it might not be possible to establish, on the other side of the border, a sort of Gretna Green for lunatics, in the shape of a house of recovery in which they might enjoy the benefits of the more advanced state of northern civilisation. It is undeniably true, as the reviewer declares, that there are in England many cases of early and slight insanity in which the law must be broken, not to save pain to the friends, but, on the highest medical advice, to save the patient's mind from becoming permanently deranged. Like all law-breaking, the course involves risks, and risks are inevitably attended by undue costliness. If such a patient, while uncertificated, could be technically said to be of unsound mind, any person who received payment for him, even if a relative, would be liable to prosecution, and to penalties on conviction; and this state of the law applies to nursing homes, and to cases of organic brain disease which may paralyse the patient. On the other hand, a relative may keep any such case uncertified if no payment be made; and it is among such that the examples of cruelty have occurred which have sometimes shocked the public. The reviewer declares it not to be well that a law which must often be broken should remain unmodified, and certainly advances cogent arguments in support of his opinion. We cannot but feel hopeful that so emphatic a declaration of the necessities of the case, in an influential publication not in the least likely to be unduly prejudiced in favour of a merely medical view, will have a definite effect in the education of the public mind and will promote the advent of changes which it declares, and shows, to be of great importance to the community.

